# Enhancing the Mechanical Properties of Biodegradable Mg Alloys Processed by Warm HPT and Thermal Treatments

**DOI:** 10.3390/ma14216399

**Published:** 2021-10-25

**Authors:** Andrea Mizelli-Ojdanic, Jelena Horky, Bernhard Mingler, Mattia Fanetti, Sandra Gardonio, Matjaz Valant, Bartosz Sulkowski, Erhard Schafler, Dmytro Orlov, Michael J. Zehetbauer

**Affiliations:** 1 Physics of Nanostructured Materials, Faculty of Physics, University of Vienna, 1090 Vienna, Austria; andrea.ojdanic@univie.ac.at (A.M.-O.); erhard.schafler@univie.ac.at (E.S.); michael.zehetbauer@univie.ac.at (M.J.Z.); 2 Faculty of Industrial Engineering, University of Applied Sciences—Technikum Wien, 1200 Vienna, Austria; 3 Center for Health & Bioresources, Biomedical Systems, AIT Austrian Institute of Technology GmbH, 2700 Wiener Neustadt, Austria; jelena.horky@ait.ac.at (J.H.); bernhard.mingler@ait.ac.at (B.M.); 4 Materials Research Laboratory, University of Nova Gorica, 5270 Ajdovscina, Slovenia; mattia.fanetti@ung.si (M.F.); sandra.gardonio@ung.si (S.G.); matjaz.valant@ung.si (M.V.); 5 Department of Material Science and Non-Ferrous Metals Engineering, Faculty of Non-Ferrous Metals, AGH-University of Science and Technology, 30-059 Kraków, Poland; sul5@agh.edu.pl; 6 Division of Materials Engineering, Department Mechanical Engineering, LTH, Lund University, 22365 Lund, Sweden

**Keywords:** severe plastic deformation (SPD), precipitates, vacancy agglomerates, magnesium alloys, biodegradability

## Abstract

In this study, several biodegradable Mg alloys (Mg5Zn, Mg5Zn0.3Ca, Mg5Zn0.15Ca, and Mg5Zn0.15Ca0.15Zr, numbers in wt%) were investigated after thermomechanical processing via high-pressure torsion (HPT) at elevated temperature as well as after additional heat treatments. Indirect and direct analyses of microstructure revealed that the significant strength increases arise not only from dislocations and precipitates but also from vacancy agglomerates. By contrast with former low-temperature processing routes applied by the authors, a significant ductility was obtained because of temperature-induced dynamic recovery. The low initial values of Young’s modulus were not significantly affected by warm HPT-processing. nor by heat treatments afterwards. Also, corrosion resistance did not change or even increase during those treatments. Altogether, the study reveals a viable processing route for the optimization of Mg alloys to provide enhanced mechanical properties while leaving the corrosion properties unaffected, suggesting it for the use as biodegradable implant material.

## 1. Introduction

Biomaterials, i.e., titanium alloys or stainless steel, are commonly used as orthopedic implants in the form of screws, needles or plates. However, subsequent surgery is needed in many cases to remove the implant after the bone tissue is healed causing additional pain to the patient [1]. Also, these implants can cause problems in the human body due to their high Young’s moduli compared to that of human bone, causing stress shielding [2]. Magnesium-based biodegradable and biocompatible alloys have been becoming increasingly important as temporary implant materials in orthopedic applications [3,4]. One of the advantages of using Mg as orthopedic implants is the low Young’s modulus, which approaches that of human bone. Besides being biocompatible, Mg is also biodegradable making it a good candidate for orthopedic implants that are absorbed by the human body after fulfilling their purpose, thus avoiding a second removal surgery [4,5]. The major drawback of using Mg is its low strength for providing stability to a damaged bone as well as its high corrosion rate in physiological environment, which may affect the mechanical properties as well as harm the tissue due to hydrogen evolution [6,7]. 

Alloying as well as applying severe plastic deformation leads to an optimization of Mg [8,9] with respect to mechanical and corrosion properties. Typical alloying elements are Ca, Zn and Zr; essential elements in the human body. Ca as well as Mg are both crucial for human bone’s formation [6,10]. Zn has a positive impact on bone healing and cell reactions [11]. Although Zr does not play a biological role, the human body contains about 250 mg of it, which makes the element biocompatible [12]. Mg alloys with Ca, Zn or Zr have good biocompatibility and biodegradability, and also possess highly favorable mechanical properties [13,14]. 

Besides alloying, mechanical properties can be further optimized by high-pressure torsion (HPT) and following heat treatment [8,9,15]. By means of dislocation and vacancy generation, HPT-processing [16] induces grain refinement and leads to a redistribution of solutes [17,18,19,20]. With additional heat treatments, the internal stresses are removed through re-distribution and loss of dislocations, this way decreasing the macroscopic strength; the generated vacancies, however, may form agglomerates which impede the dislocation movement and thus increase the macroscopic strength [8,21]. Moreover, nano-scale intermetallic precipitates form at special ageing conditions, as was shown by Orlov et al. for the ZK60 Mg alloy [22]. Also Mima and Tanaka [23] found three important low-temperature ranges for MgZn systems: (i) below 60 °C stable Guinier–Preston (GP) zones form; (ii) in a temperature range of 60–110 °C stable rod-type and basal platelet-type precipitates form along with unstable GP zones; and (iii) above 110 °C stable rod-type and basal platelet-type precipitates form, where the rod-type are the most stable ones [24,25]. 

As already reported, a proper processing method has been explored for the optimization of Mg alloys by Horky et al. [8] and Ojdanic et al. [9,15] with the compositions (in wt%) Mg0.2Zn0.5Ca and Mg0.6Zn0.5Ca [8], and Mg5Zn0.3Ca, Mg5Zn0.15Ca, Mg5Zn0.15ca0.15Zr, and Mg5Zn [9,15]. Having processed all these materials by a special processing route, i.e., annealing at 350–400 °C, subsequent furnace cooling to room temperature (RT) through 12–24 h, then followed by HPT-processing by 0.5 rotation at RT, and additional heat treatment at 100 °C for 24 h, show samples with a very high strength increase of up to 250%, but with a high brittleness.

Therefore, in the current study, the processing method for the named alloys was to optimize them in order to achieve better results regarding the combination of strength, ductility and corrosion. The thermal processing was changed to thermomechanical processing, by means of HPT-processing to high strains at elevated temperature and subsequent heat treatments. Except for hardness, tensile strength and Young’s modulus, also ductility and corrosion properties in simulated body fluid (SBF) have been in focus of this work. The microstructure was analyzed in detail by electron microscopy, differential scanning calorimetry and X-ray line profile analysis [26,27]. 

## 2. Experimental Procedure

### 2.1. Materials and Sample Preparation

Four Mg alloys were studied with compositions Mg5Zn, Mg5Zn0.3Ca, Mg5Zn0.15Ca, and Mg5Zn0.15Ca0.15Zr. The alloys were cast at the LKR Leichtmetallkompetenzzentrum Ranshofen, a subsidiary of AIT Austrian Institute of Technology. The chemical composition of the alloys is shown in Table 1.

### 2.2. High-Pressure Torsion (HPT)-Processing

Disc-shaped samples with a diameter of 10 mm and thickness of 0.7 mm were machined, for the sake of HPT-processing. HPT was achieved by applying a hydrostatic pressure of 4 GPa, and a rotation speed of 0.3 rot/min. The samples have been deformed to shear strains:

(1) $$\gamma_{T}=\frac{2\pi Nr}{h}$$

which becomes smaller by a factor 1/√3 when converted to a corresponding von-Mises strain ε_vM_ [16]. Here, *N* means the number of rotations, *r* the samples’ radius, and *h* the sample thickness. HPT- processing was carried out at 285 °C and 10 rotations (i.e., ε_vM_ = 330) in order to produce a supersaturated solid-solution condition (SSSS)—a condition completely free from primary precipitates in casting. 

### 2.3. Heat Treatments

Heat treatments of the HPT-processed samples were executed for different time periods up to 48 h, and at different temperatures between 60 °C and 150 °C in a silicon oil bath with a temperature variation under ±0.5 °C. Then the samples were quenched in water having room temperature (RT) to ensure fast cooling and accurate heating periods [9].

### 2.4. Characterization of Microstructure and Properties

#### 2.4.1. Mechanical Properties

For reliable tests of microhardness, the samples were polished by 1200-grit SiC paper and cleaned with ethanol, in order to minimize oxidation and to obtain a defined surface condition.

Vickers hardness (HV0.05) was measured with an ANTON PAAR MHT-4 microhardness tester, by applying a load of 0.5 N for 10 s. The indentation area was analyzed within a Light Microscope ZEISS 20 AXIOPLAN, by measuring the indents’ diagonal lengths on images obtained from a CCD camera. Their average was not affected by any asymmetry of indents, and crack formation was not observed at all. For determination of the microhardness of an HPT-processed sample, the indents were distributed over the whole cross section, by applying them along full radii of the disc-shaped samples. Only indents with the same radius, i.e., distance from the center of the HPT disc, were averaged. At least 10 indents per sample state were evaluated. 

The Young’s modulus was measured by a microindentation tester ANTON PAAR MHT. At least 20 indents per sample state were carried out. 

**Figure 1:**
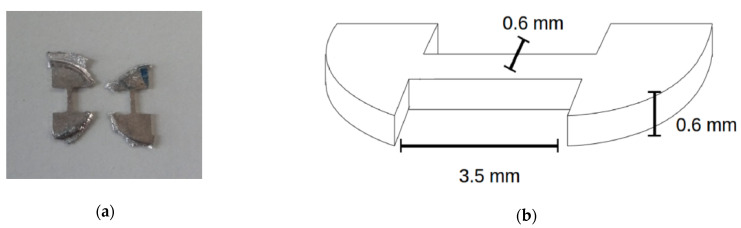
(**a**) Tensile test samples cut from Mg alloys in question, (**b**) dimensions of a cut sample [15].

Strength and ductility were investigated by tensile testing. Dogbone-shaped specimens with a cross-section of 0.6 × 0.6 mm^2^ and a parallel gauge length of 3.5 mm on average (Figure 1) were cut by spark erosion from the HPT-processed/heat treated discs. The tensile samples were cut at a position of approximately 3 mm off the center of the HPT-discs. The tensile tests were performed by a micro-tensile low-load machine MESSPHYSIK suitable for measurements of tensile properties of small-scaled samples. Forces of up to 120 N at a strain rate of approximately 1 × 10^−3^ s^−1^ were applied. To obtain reliable values for the tensile properties, at least three samples with the same preparation history were tested and averaged.

#### 2.4.2. Electron Microscopy

In order to investigate the grain structure and the distribution of precipitates at least at the microscale, scanning electron microscopy (SEM) in backscattered-electron (BSE) imaging mode was performed. For this purpose, a ZEISS SUPRA 55 VP SEM equipped with an energy-dispersive X-ray spectroscopy (EDX) analysis and imaging system was used. Samples were mechanically ground and polished by means of an ethanol lubricant and a 0.05 µm alumina suspension. Evolutions of the microstructure on the nano-scale were investigated by transmission electron microscopy (TEM) and scanning transmission electron microscopy (STEM), using a JEOL JEM 2100F-UHR equipped with annular dark field detector. The specimens for TEM/STEM analyses were prepared as reported by Ojdanic et al. [15].

#### 2.4.3. Differential Scanning Calorimetry

The measurements differential scanning calorimetry (DSC) were carried out with two instruments, a NETZSCH DSC204 and a PERKIN ELMER DSC8500. Heating was performed in a temperature range from 25 to 450 °C with a heating rate of 10 K/min. During DSC measurements, the occurrence of exothermic peaks indicates the annealing of deformation-induced defects while endotherm ones represent phase transformations. All the DSC peaks were identified by comparison with a base line obtained in a second DSC run with the same sample. The area of each peak represents the formation enthalpy of the annealing defect, from which its density can be evaluated. For dislocations—in a first-order approach—the stored energy $E_{disl}\;$of a dislocation corresponds to its self energy i.e., mainly to its strain field [28]. The inner cut-off radius is approximately equal to the Burgers vector (*b* = 0.32 nm), the outer cut-off radius can be taken as the distance to the neighbouring dislocation, especially when—like in the current case of HPT deformation—the dislocations are part of cell/subgrain boundaries with screened strain fields [28]. $E_{disl}\;$is then related to the dislocation density *ρ* as:

(2) $$E_{disl}=Gb^{2}\frac{\rho }{4\pi \kappa }\times \ln\left({\left(b\sqrt{\rho }\right)}^{-1}\right)$$

Here, *G* is the shear modulus (*G* = 17 GPa). *κ* denotes the arithmetic average of 1 and (1 − ν), with ν = 0.343 as the Poisson ratio, assuming equal parts of edge and screw dislocations. This first-order approach neglects not only the core energy but also any interaction energy; thus it represents a lower limit of dislocation stored energy, and an upper limit of dislocation density.

By a similar first-order approach, the vacancy concentration *c_v_* can be evaluated from the stored energy of vacancies *E_vac_* and the formation enthalpy per vacancy Δ*H*, by means of Equation (3) (for Mg, Δ*H* = 1.27 × 10^−19^ J = 0.79 eV [29]):

(3) $$c_{V}=\frac{E_{vac}}{∆H\times \chi \times N_{a}}$$

with χ being the mole fraction, and *N_a_* the Avogadro’s number.

Information on the type of the defect can be gained from the peak temperature *T_max_* and the activation (defects’ migration) enthalpy *Q*. The latter can be determined by the method of Kissinger [30] by evaluating the shift of the peak temperature while varying the heating rate. By using,

(4) $$\ln\left(\frac{\phi }{T^{2}}\right)=-\frac{Q}{R}\frac{1}{T_{max}}+const$$

for different heating rates *Φ* and peak temperatures *T_max_*, the activation enthalpy *Q* can be calculated; *R* means the gas constant.

#### 2.4.4. X-ray Diffraction Peak Profile Analysis (XPA)

For the determination of dislocation density *ρ*, selected Mg samples have been subjected to X-ray diffraction peak profile analysis (XPA). As the X-ray source, a RIGAKU MM9 X-ray rotating anode generator was used providing monochromatic Co-K*_α_* radiation, corresponding to a wavelength λ = 1.79 nm. The peak profiles were taken by a curved positioned sensitive detector of the type INEL CPS-590, spanning an angular range of 90° between 40° and 130° For understanding Bragg diffraction peak broadening in terms of dislocation density and crystallite size, see [26,27]. For the evaluation of dislocation density, the open source software (C)MWP-fit [27] was used.

#### 2.4.5. Texture Analysis

Texture investigations were carried out with a BRUKER D-8 DISCOVER X-ray diffractometer equipped with a GADDS (general area diffraction detection system) area detector. The area detector can cover up to 35 ° of 2θ range at one fixed position. A copper anode was used for the radiation of X-rays at 40 kV and 40 mA, with a wavelength of 0.154 nm for CuKα_1_. X-ray texture measurements were performed by the reflection method, which is based on four main reflections in Mg used for the orientation distribution function (ODF), which was calculated by the Arbitrary Defined Cells (ADC) method with LaboTex3.0 texture analysis software (for details see [15]).

##### 2.4.6. Corrosion Tests

The corrosion rate of the Mg5Zn alloy was measured by immersing samples in simulated body fluid (SBF27 from [31] with Tris-HCl buffer) at a human body temperature of 37 °C for a period of three weeks and collecting the evolving hydrogen gas. Always two discs of each tested condition were immersed into 250 mL of SBF. The initial pH value of the SBF was 7.35 and increased to values between 8.2 and 8.8 within a week, being higher in the case of samples with a higher corrosion rate. After each week, the SBF was replaced to restore the initial pH value and ion concentration. Two experiments were performed for each tested condition to obtain a mean value and a standard deviation. More details of the corrosion measurements can be found in [15].

## 3. Results

### 3.1. Effects of Severe Plastic Deformation at Elevated Temperature

**Figure 2:**
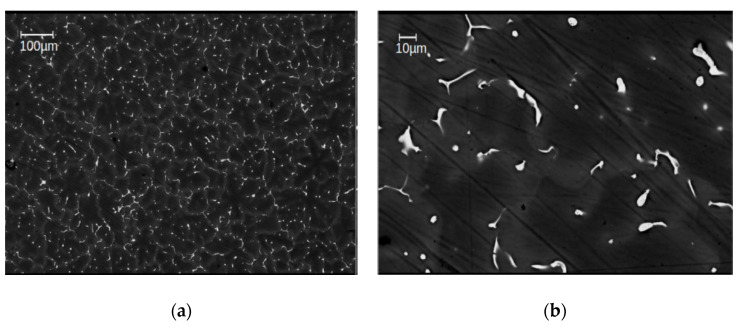
Scanning electron microscope (SEM) images of the as-cast alloy Mg5Zn0.3Ca, with low (**a**) and high (**b**) magnification [15].

**Figure 3:**
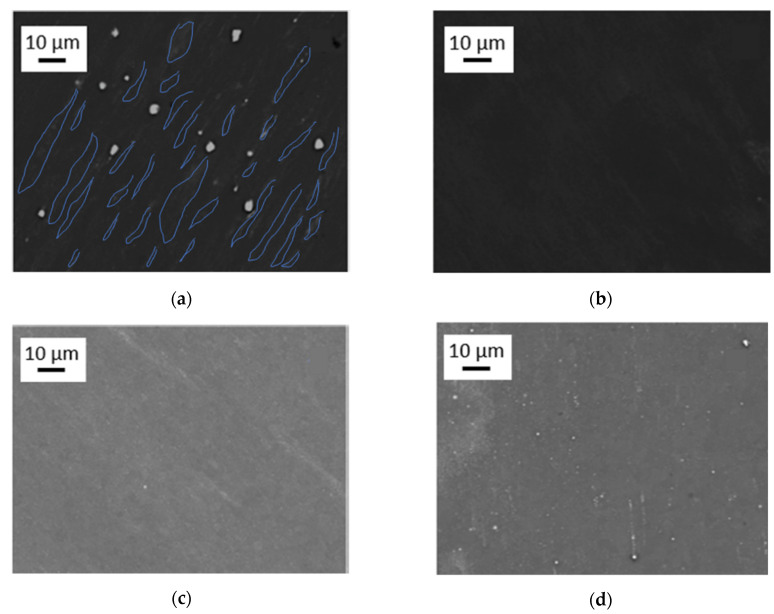
SEM images of high-temperature high-pressure torsion (HPT)-processed samples at 285 °C, 4 GPa and 10 rotations, taken from areas with γ_T_ ~ 320, for the materials (**a**) Mg5Zn0.3Ca, (**b**) Mg5Zn, (**c**) Mg5Zn0.15Ca, and (**d**) Mg5Zn0.15Ca0.15Zr. In (**a**), the grain area contrasts have been visualized by blue lines.

From EBSD and BSE investigations done in all materials studied in this work, the grain size of the as-cast samples has been evaluated to be 49 ± 5 µm in average (Figure 2), after HPT-deformation at 285 °C as between 10–40 µm, i.e., 25 ± 3 µm on average (Figure 3a). That means that in spite of the severe plastic deformation processing, the grain size remained close to that of the as-cast materials i.e., within about a factor of 2. As concerns the precipitates, SEM images of the as-cast alloys show a huge number of primary ones with a size of 5–10 µm that are distributed homogeneously in each of the sample conditions (Figure 2). The total volume fraction of primary precipitates was determined to be approximately ~2%, as estimated from the total area of the particles by means of standard image analysis methods. A detailed EDS analysis on primary precipitates can be found in [15].

HPT processing at 285 °C, the primary precipitates become thermally and mechanically re-solutionized. The SSSS-condition could be achieved for all investigated alloys (Figure 3), except for Mg5Zn0.3Ca, which still showed occasional residual primary precipitates (Figure 3a). Nevertheless, for all Mg alloys including the latter, the volume fraction of primary precipitates could be decreased to less than 1%.

**Figure 4:**
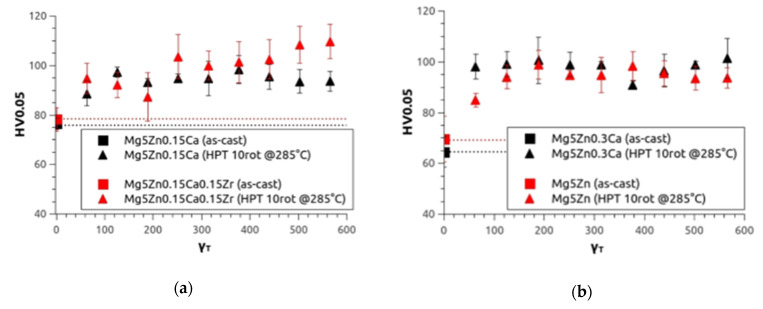
Microhardness results of (**a**) Mg5Zn0.15Ca (black) and Mg5Zn0.15Ca0.15Zr (red), (**b**) Mg5Zn0.3Ca (black) and Mg5Zn (red) in the as-cast condition (dotted line) and after HPT deformation at 4 GPa, 10 rotations at 285 °C as a function of the torsional strain.

As in the first investigation, microhardness (HV0.05) measurements were taken. In Figure 4, the microhardness values are presented as a function of torsional shear strain γ_T_ calculated by means of Equation (1). The values increase with increasing γ_T,_ and finally reach saturation at about HV0.05 = 100 for all four alloys. Starting from the as-cast condition, the samples show an increase of microhardness by 35 % for Mg5Zn0.15Ca, 25 % for Mg5Zn0.15Ca0.15Zr, 55 % for Mg5Zn0.3Ca, and 42 % for Mg5Zn.

### 3.2. Effects of Isothermal Heat Treatments

**Figure 5:**
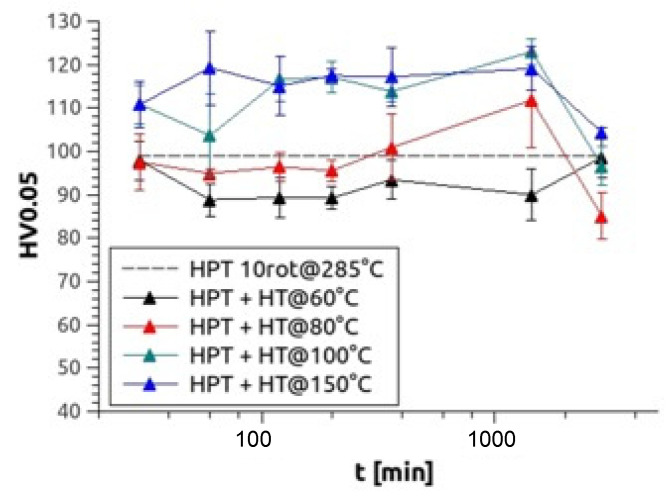
Microhardness results of Mg5Zn0.3Ca, HPT-processed at 4 GPa and 10 rotations at 285 °C and heat treated at 60 °C (black), 80 °C (red), 100 °C (green) and 150 °C (blue) up to 48 h.

After HPT processed at elevated temperature, some samples were also heat-treated for up to 48 h at temperatures between 60–150 °C. During the heat treatments, no changes in the grain size/size distribution were obtained which was present immediately after the warm HPT processing. Exemplarily for Mg5Zn0.3Ca, Figure 5 demonstrates the effects of various heat treatments on microhardness, showing a significant hardness peak at 100 °C after 1440 min (24 h) of heat treatment and a similar high peak at 150 °C after 120 min (2 h) of heat treatment. For all alloys, a hardness increase of around 25% could be observed at 100 °C and around 20% at 150 °C (Table 2).

### 3.3. Tensile Strength and Ductility

**Figure 6:**
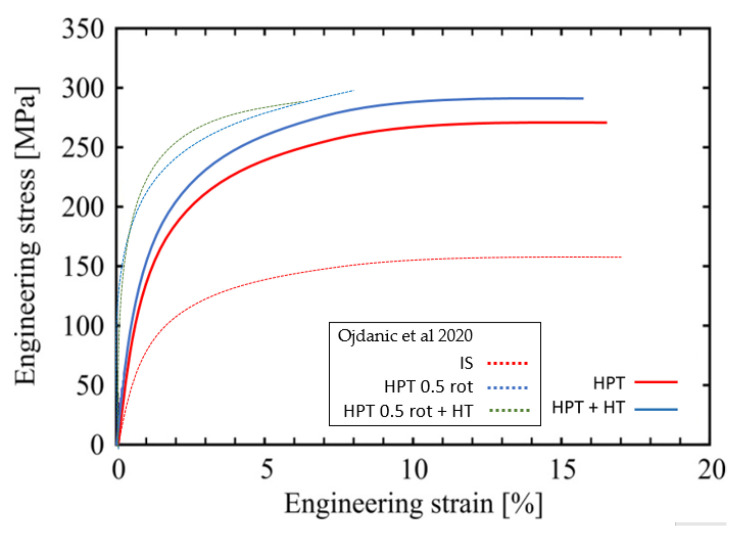
Representative tensile stress-strain curves for Mg5Zn0.15Ca, HPT-processed (10 rotations at 285 °C) (full line—red) and after additional heat treatment at 100 °C for 24 h (full-line blue). For comparison, the dotted lines show the results of the previous study [15] of the same alloy processed as follows: annealing at 450 °C for 24 h and then furnace cooled to RT (IS), HPT-processed at 4 GPa and 0.5 rotations at RT (HPT 0.5 rot), and HPT plus heat treatment at 100 °C for 24 h (HPT 0.5 rot +HT).

Tensile tests on all four alloys were conducted on as-cast material, HPT deformed, and additionally heat treated for 24 h at 100 °C. All tests were performed until failure, which occurred close to the center of specimens. Typical engineering stress–strain curves obtained by micro-tensile testing are presented in Figure 6. Table 3 presents values of the yield strength determined at 1% of plastic strain, as well as those of the ultimate tensile strength (UTS). The post-HPT heat treatment led to marked increases in yield strength of up to ~20% for Mg5Zn0.15Ca0.15Zr, while Mg5Zn0.3Ca and Mg5Zn0.15Ca showed a slight decrease. The latter was shown also by the values of ultimate tensile strength for the same alloys. However, the values of elongation to failure which still reached up to 19 % after HPT were not significantly affected by the thermal treatment after HPT-processing. The average values for strength and ductility obtained from tensile tests are summarized in Table 3.

### 3.4. Evolution of Texture and Young’s Modulus

**Figure 7:**
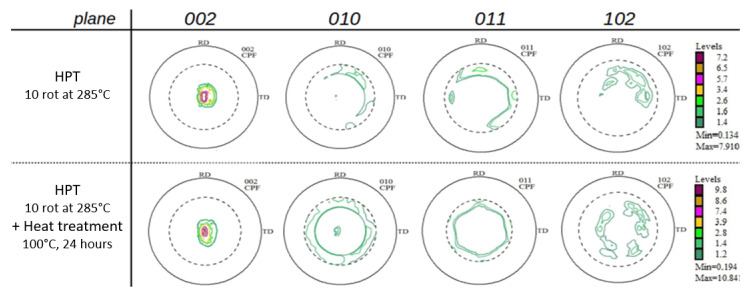
Pole figures displaying crystallographic textures of Mg5Zn0.15Ca after various treatments.

Pole figures (002) (010) (011) and (102) of the samples were determined, after HPT-processing as well as after additional thermal treatment (Figure 7). While the textures after low-temperature HPT-processing found in our previous paper [15] are typical shear textures of Mg and Mg-rich alloys [32,33] the current textures not only comprise shear deformation components but also some features of dynamic recrystallization (after HPT, upper raw). As already observed with the low-temperature HPT-processed samples [15], thermal treatment at 100 °C leads to a randomization of the texture, as a consequence of static recrystallization. Such effects have been already observed by us [32] and other authors [33] pioneering the textures of severely deformed Mg and Mg alloys.

Values of Young’s modulus E of all materials and treatments were evaluated from microhardness indentation tests. After HPT-processing, they all were between 31–42 GPa (Table 4) with errors of about ± 2 GPa. The thermal treatment at 100 °C did not change the Young’s modulus significantly, i.e., were close to the measuring error. In order to substantiate these results, we tried to simulate the magnitude of Young’s modulus and its changes due to the thermal treatment at 100 °C, at least for the materials Mg5Zn0.3Ca and Mg5Zn0.15Ca. For these simulations, the procedures described in detail in paper [15] have been applied. Again, the changes due to thermal treatment were negligible. As in [15], the constant positive offset of the simulated values of E compared to the experimental ones may have arisen from the upper-limit approach of the calculation as well as from the neglect of constituents Zn and Ca therein.

### 3.5. Electron Microscopy Analysis of Precipitates

For TEM analyses the alloy Mg5Zn was investigated in the following conditions:

(1) HPT-processed at 4 GPa for 10 rotations at 285 °C;
(2) HPT-processed at 4 GPa for 10 rotations at 285 °C and heat treated at 100 °C for 24 h;
(3) HPT-processed at 4 GPa for 10 rotations at 285 °C and heat treated at 150 °C for 2 h.

**Figure 8:**
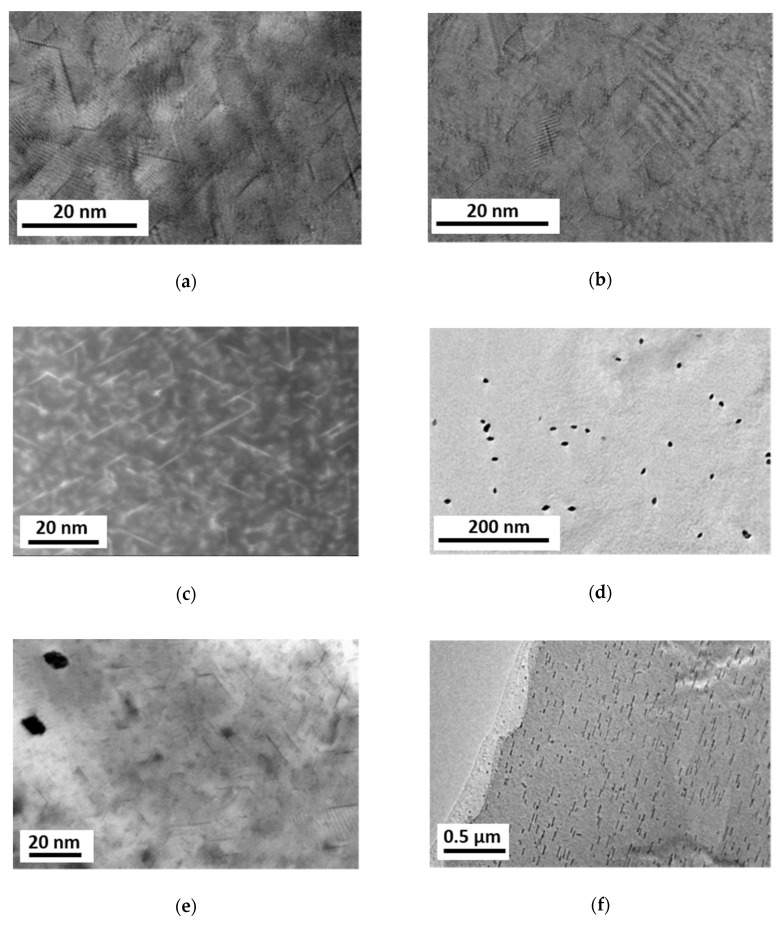
Micrographs revealing precipitate structures in Mg5Zn samples: (**a**) bright-field transmission electron microscopy (TEM) after HPT-processing; (**b**) bright-field TEM and (**c**) HAADF STEM after HPT and following heat treatment at 100 °C for 24 h; (**d**–**f**) bright-field TEM after HPT and following heat treatment at 150 °C for 2 h. In the micrographs (**a**–**e**), the observation is close to <0001>, while in (**f**) it is close to <10$\bar{1}$0>.

Although the primary precipitates were eliminated through the HPT processing, small prismatic precipitates could be found in all material states having lengths between 5–20 nm, thicknesses less than 1 nm and orientations along three <a> directions at 120°/60° to each other. The prismatic platelets show homogeneous distributions in the as-HPT-processed as well as in additionally heat treated at 100 °C samples (conditions (1) and (2), Figure 8a–c). The sample heat treated at 150 °C (3) showed not only prismatic but also rod-like precipitates aligned in <0001> directions, Figure 8d–f. The rod-like precipitates have been formed far away from the prismatic precipitates, Figure 8e, also in much smaller densities.

### 3.6. Determination of Severe Plastic Deformation (SPD)-Induced Defect Densities by Differential Scanning Calorimetry (DSC) and XPA

**Figure 9:**
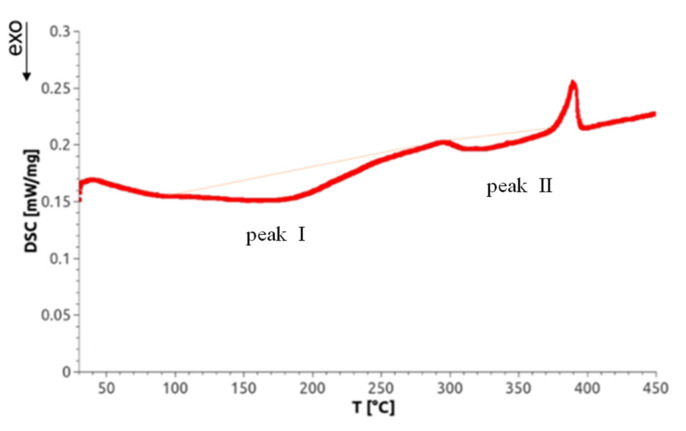
A representative heat flow curve as function of the temperature, for warm HPT-processed Mg5Zn0.3Ca exhibiting two exothermal peaks I and II. The thin red line is qualitatively similar to the base line which has been recorded in a second differential scanning calorimetry (DSC) run (for details see Section 2.4.3).

Differential scanning calorimetry not only allows for measurement of phase transitions but also can detect lattice defects through selective annealing. Concerning SPD-processed materials, Setman et al. [18] identified—as a function of annealing temperature—three annealing peaks on HPT-processed Ni (99.998% purity): (i) from the annealing of single and/or double vacancies, (ii) from vacancy agglomerates, and (iii) from dislocations. Because the temperature of the latter often depends on the deformation strain applied, the annealing peaks of the latter two defects can overlap. As a representative for the DSC scans of all Mg alloys investigated, Figure 9 shows a scan of an HPT-processed Mg5Zn0.3Ca sample. There appear two exotherm peaks that are called ‘peak I’ and ‘peak II’. At about 400 °C, a sharp endotherm peak emerges which indicates the occurrence of a phase transformation, probably the dissolution of precipitates found by TEM as indicated in Section 3.5. Beyond this temperature, the microstructure of original as-cast material is reached so that the DSC mediated defect analysis was stopped.

The stored energies E_total_ of the HPT-induced defects given in Table 5 (peak I) and Table 6 (peak II) are evaluated from the areas of the peaks, after having averaged them from 4 independent experiments. The error given represents the maximum deviation.

From the measured stored energies E_total_ of the defects, their concentrations can be determined by means of Equations (2) and (3). Because of the possible overlap of peaks, however, for the determination of the real densities of vacancies, it was the best way to follow a procedure already applied in [18] and in the previous paper [15] 15 of the authors. Here, from the measured dislocation density ρ, the stored energy of dislocations E_disl_ was calculated via Equation (2) and subtracted from the total stored energy E_total_, with the stored energy left to be attributed to the vacancy-type defects, E_vac_, thus yielding, via Equation (3), the vacancy concentrations c_v_. Those, together with the dislocation densities measured by XPA, are presented in Table 5 and Table 6 for both peaks for all materials studied.

**Figure 10:**
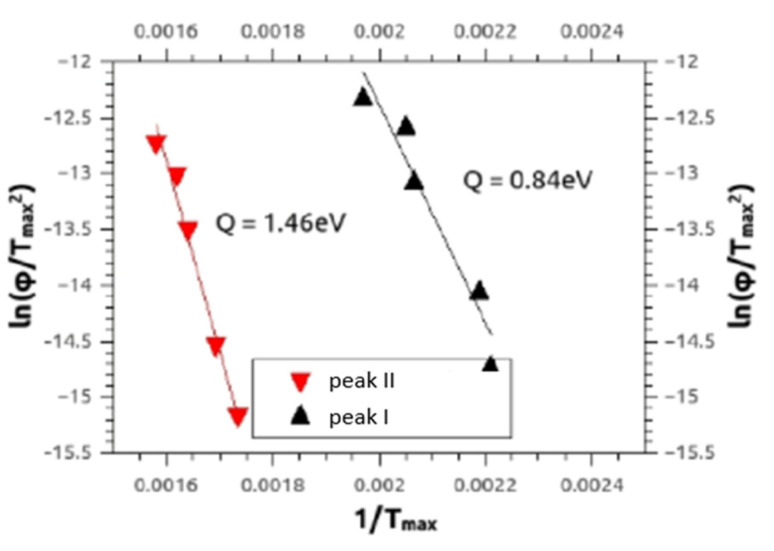
Typical Kissinger plots referring to Equation (5) for peak I and peak II measured by DSC, for HPT-processed Mg5Zn at 4 GPa for 10 rot. at 285 °C. The full lines represent the regressions to the experimental data, from which the activation enthalpies Q given were calculated.

Similar to our paper [15] DSC also provided the defect migration enthalpies Q (Figure 10) being characteristic of the type of the annealing defect, by means of the Kissinger–Ozawa method [30]. The results for activation enthalpies of peak I and peak II were between 0.6–1.5 ± 0.1 eV, and between 1.3–3.4 ± 0.3 eV, respectively, for all alloys Mg5Zn0.3Ca, Mg5Zn, Mg5Zn0.15Ca and Mg5Zn0.15Ca0.15Zr.

### 3.7. Corrosion Tests

**Figure 11:**
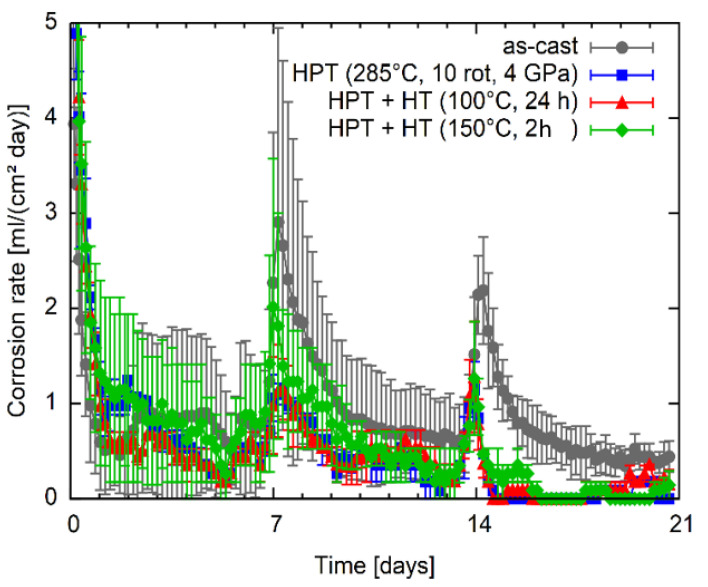
Corrosion rate of the Mg5Zn alloy in as-cast, HPT-deformed as well as subsequently heat-treated conditions as a function of the immersion time.

Results of the immersion tests of Mg5Zn in simulated body fluid at human body temperature are shown in Figure 11. The corrosion rate is presented as a function of immersion time. It can be seen that the corrosion rate is highest directly after the start of the test. After each renewal of the SBF peak-link increase in the corrosion rate was observed. Although the results have an apparent scatter, it can be seen that except for in the first days, HPT processing at 285 °C leads to a clearly lower corrosion rate compared to the as-cast condition. The subsequent heat treatment at 100 °C does not significantly change the corrosion rate. When analyzing the results for the samples heat treated at 150 °C after HPT processing; however, a slight tendency to higher corrosion rates could be observed, especially during the second week of testing.

## 4. Discussion

### 4.1. The Effects of Warm HPT Processing and Additional Heat Treatments

Learning from the deficiencies of materials studied in our previous work [15], two goals were followed in the present one, i.e., to (i) entirely re-solutionize the primary precipitates which remained from casting, to (ii) increase the ductility without compromising high strength values, and to (iii) leave other beneficial properties such as low Young’s modulus and corrosion rate unaffected. We tried to reach these goals by increasing the HPT processing temperature to 285 °C of all four alloys, applying 4 GPa and 10 rotations (i.e., ε_vM_ = 330). The temperature 285 °C was chosen in order not to exceed the Scheil solidus (294 °C) thus avoiding incipient melting [34,35]. As in [15], additional isothermal treatments at T = 100 °C and 150 °C were carried out to further approach the goals (i) and (ii).

Concerning goal (i), indeed the primary precipitates could be eliminated in all alloys except Mg5Zn0.3Ca where at least their volume fraction remained below 1% (Figure 3).

As for goal (ii), increases of microhardness of up to 55 % were achieved by the warm HPT-processing, not at least thanks to the very high strains being 2.5 times larger compared to those of low-temperature HPT-processing in [15]. Furthermore, additional strength increases of 25% and 20% occurred due to the subsequent isothermal heat treatment after HPT, at T = 100 °C and 150 °C, respectively, followed by a decrease after 24 h and 2 h, respectively, reflecting a kind of over-aging process. All the strength increases are not only seen in microhardness but also in tensile tests either in yield stress or ultimate tensile strength. Above all, the latter show the significant increase of ductility of the current warm HPT processing and/or thermal treatments compared to the low-temperature HPT processing/thermal treatments reported in the papers [8,15] Figure 6 and Table 4, being now at least 16–19% instead of about 5% previously.

Also goal (iii) could be reached with the warm HPT processing and thermal treatments: nor did the rather low initial Young’s modulus of about 40 GPa of all the Mg alloys investigated change after warm HPT processing or after additional heat treatment. The same is true for the corrosion rate except the materials heat treated at 150 °C after HPT processing, where some tendency of increase was observed.

### 4.2. Reasons for the Effects of Warm HPT-Processing and Additional Heat Treatments

First, we wish to clarify where the observed ***increases in strength*** (both microhardness and yield stress/UTS) come from. During warm HPT processing, the grain size decreased by about a factor of 2 within the range 10–100 µm. In fact, the average grain size decreased from 49 µm to 25 µm which causes—according to a recent review on Hall–Petch relationships in Mg alloys ([36], see Figure 6 therein)—an increase of about 7% of the flow stress, which is only 10% of the total HPT-induced flow stress (compare Figure 6 of this work); the additional heat treatments showed no detectable change in grain size at all, so that—in a search of the main sources of strength increases—we focused on other reasons. These are: (i) HPT-generated dislocations, (ii) HPT-induced formation of precipitations, and/or (iii) hardening from HPT-generated vacancy agglomerates, as has been shown for Mg alloys by Horky et al. [8] and Ojdanic et al. [15].

Discussing first the effects of ***warm HPT-processing***, the formation of precipitates (ii) can be ruled out as the TEM observations showed no differences—either in the type or density—of precipitation microstructure. Precipitates can only contribute to macroscopic strength being already present before HPT-processing treatment; according to our investigations reported in papers [9,15] its contribution may amount 20% at maximum. However, considering the dislocation densities produced by HPT-processing at low and high temperatures, both amounted to about ρ≅ 6.3 × 10^14^ m^2^, not least due to the large deformation applied in case of high temperature deformation, balancing the generally lower dislocation densities produced at the higher deformation temperature. Assuming the interaction/configuration parameter of dislocations α = 0.1 in highly deformed microstructures, as part of the Taylor equation:

(5) $$∆H=m\;\sigma =m\;M\;∆\tau =m\;M\;\alpha \;G\;b\;\sqrt{\rho }$$

with *M* = 4.2 as the Taylor factor, *m* = 4.2 Tabor’s factor, G and b as shear modulus and Burgers vector, respectively (values see Equation (2)), a value of ∆H = 294 MPa results which just fits to the missing 80 % of the measured total increase of strength due to work hardening. Because of the higher deformation temperature in warm HPT processing, however, α = 0.08 may be more realistic because of a more recrystallized microstructure compared to that of low-temperature HPT [15], with less internal stresses and the extended ductility observed. Using (5) with α = 0.08, the dislocation-caused strength becomes smaller and, with regard to the contribution of grain refinement being about 7% (see estimation in first paragraph of this section), the precipitation part is responsible for at least 20% of the total strength.

Now let us consider reasons for the further hardening observed during additional heat treatment at 100 °C and 150 °C. The reason (ii) was that precipitation hardening is not relevant as neither additional nor a new type of precipitate could be observed by TEM after 100°C aging; on the other hand, the new type of precipitates observed by TEM at 150 °C aging seems to be not important because no hardening but softening effects were found at this aging temperature. Considering reason (i) dislocation hardening, this can be ruled out as the dislocation density did not increase during additional heat treatment, and rearrangements of dislocations can only lead to softening rather than hardening. Therefore, as argued in our previous works [8,15], it is only the agglomeration of vacancies that can lead to hardening during the heat treatment. An estimation can be done by means of Kirchner’s model [37]:

(6) $$\Delta \sigma =\frac{Gb}{k}N^{a}d^{3a-1}$$

Here, *N* means the loop density (number of loops/m^3^), d the average loop diameter, and *a* and *k* constants which depend on the ratio of loop distance (λ = *N*^−1/3^) to diameter. For a ratio λ/*d* > 10, the constants are equal to *a* = 1/2 and *k* = 0.122, otherwise *a* = 4/3 and *k* = 0.001. Equation (6) shows that the strengthening of loops becomes stronger when the loop density is larger.

**Figure 12:**
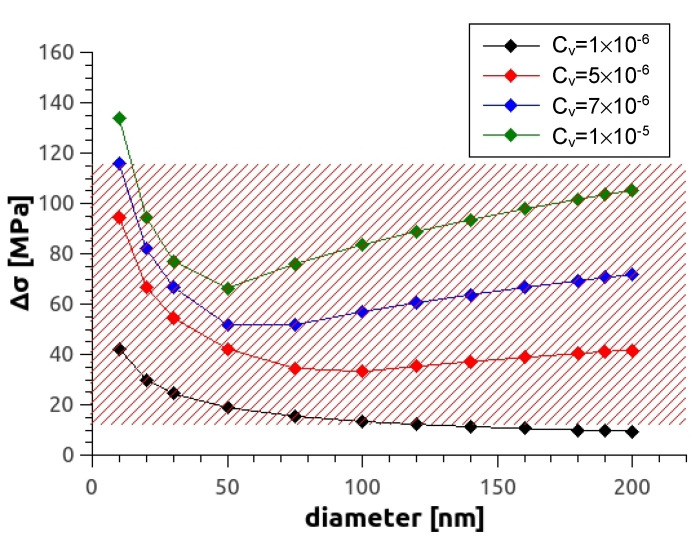
Increase of strength as a function of loop diameter at various vacancy concentrations. The red shaded area represents values of possible vacancy-induced strength in investigations [8,15] and the current one.

The amount of loop hardening in Kirchner’s equation can be estimated by inserting the determined values for the vacancy concentration from Table 5, and by an assumption for the loop diameter *d* (10–100 nm [38]). For the loops’ Burgers vector, *b* = 0.32 nm was taken. The number of vacancies per loop (vac_loop_), and the loop density *N* assuming circular loops are given by:

(7) $$vac_{loop}=\frac{d^{2}\pi }{4b^{2}}$$

(8) $$N=\frac{c_{V}}{vac_{loop}}$$

where *c_v_* is the vacancy concentration assuming that all of them form loops. The calculated dependence of theoretical yield strength on average vacancy concentration for different loop sizes is shown in Figure 12. For the alloy Mg5Zn0.3Ca studied in this work, it can be seen that according to the model, a vacancy concentration of the order *c_v_* ≅ 10^−6^ is already sufficient to account for the observed hardening of ∆σ = 14–34 MPa, with loop sizes being between 15–50 nm.

For the alloys investigated in this paper, the measured generated vacancy concentrations amount to typically *c_v_* ≅ 10^−4^, and one wonders why the vacancy hardening is not much larger than observed. The answer is two-fold: (i) at the aging temperatures 100–150 °C, according to the DSC plot, vacancies not only agglomerate but also start to anneal, and (ii) not all vacancies that resist annealing are part of agglomerates and stay single, as already reported from similar simulations in the previous study [15].

The fact that the hardness peak at 100 °C after 24 h of heat treatment is caused by vacancy agglomeration [18] is confirmed by the DSC measurements, which show peak I (Figure 9) to start already at this very temperature. The number of dislocations that also anneal at this temperature, however, is about 25% higher than in the case of low-temperature HPT-processing described in our paper [15]; this indicates that already at this temperature some primary recrystallization has taken place, which is in accordance with the special features of textures of warm-HPT presented in Section 3.4 of this paper. It is also well known that microstructures during and/or after recrystallization are poor in internal stresses compared to those immediately after plastic deformation; this explains why the ductility of warm HPT-processed materials is much larger (3×, until about 15%) than in the case of low-temperature HPT-processed ones (for the consequences to the dislocation configuration parameter α, see above).

The present Kissinger analyses are in line with the above interpretation of annealing processes. Concerning peak I, they show—all materials included—activation enthalpies Q(I) between 0.6–1.5 eV, which agrees with literature values of vacancy migration enthalpy for Mg and Mg alloys, being 0.8–1.0 eV [29]. The evaluated Q(I) values, however, extend to 1.5 eV, which indicates that dislocations may start to anneal already within peak I. The enthalpies Q(II) evaluated for peak II are—in any case—in accordance with those of dislocation annealing/primary recrystallization as they equal to values between 1.3 and 3.4 eV. The average error for both values is ±0.3 eV.

When comparing the stress–strain curves of the alloys after HPT-processing at 10 rotations at 285 °C and those additionally heat treated at 100 °C for 24 h (Figure 6), it can be seen that ductility is not affected by the thermal treatments. Compared to the processing method of the previous study, it is obvious that already the samples’ HPT processed at 285 °C by 10 rotations are much more ductile than samples HPT processed at RT by 0.5 rotations. The brittleness of the latter samples may be caused by the formation of a large number of vacancy agglomerates along with immobile dislocations. Anyway, in the current study, the vacancy concentration and therefore the concentration of vacancy agglomerates is much lower (about one order of magnitude [15]).

As concerns the effects of warm HPT processing and/or thermal treatment on the Young’s modulus, for all materials, the microindentation measurements of Young’s modulus E showed moderate differences of E by 11 GPa between the HPT-processed and additionally heat treated conditions, and by only 4 GPa at maximum within the same material (Table 4). Compared to the E-values after low- temperature HPT, and after HPT plus heat treatment [15], the E-values in this work are generally lower, which may be attributed to texture features typical of dynamic recrystallization (higher processing temperature) and static recrystallization (annealing treatment), compare Figure 7 [32,33]. In general, E stays low for all alloys i.e., it does not exceed 42 GPa which is still close to that of bone (E = 10–30 GPa) [39]; thus the conditions to avoid the stress-shielding effect in implant applications [2] are still fulfilled.

### 4.3. Corrosion Behavior during Processing

It is known that precipitates can strongly influence the corrosion behavior of Mg alloys [40]. Most precipitates are more noble than the Mg matrix and, therefore, increase the dissolution of the material; however, depending on their number and distribution in the material also other effects can occur [41,42]. In the case of Mg5Zn alloy under investigation in this work, the dissolution of primary precipitates by the warm HPT-processing was achieved, which results in the decrease of corrosion rate as shown in Figure 11. The high number of dislocations generated during the warm HPT-processing does not seem to affect the corrosion rate. Subsequent heat treatments applied on the HPT-processed material increase the hardness and strength by the generation of vacancy agglomerates, not by the formation of precipitates. As the corrosion rate shows no significant change, it seems that the agglomeration of deformation induced vacancies—like the dislocations—do not increase the corrosion rate.

## 5. Summary and Conclusions

In the present study, new routes for the improvement of various MgZnCa(Zr) alloys with respect to their mechanical and corrosion properties were investigated. Two ternary alloys (Mg5Zn0.3Ca and Mg5Zn0.15Ca), one quaternary (Mg5Zn0.15Ca0.15Zr) and one binary alloy (Mg5Zn) were studied via measurements of hardness, Young’s modulus, tensile strength and of the corrosion rates; in parallel numerous microstructural investigations were undertaken by applying methods of optical and electron microscopy, and especially of X-ray diffraction and DSC to analyze the nature and density of lattice defects involved. The alloys were processed by warm HPT for 10 rotations (von Mises equivalent strain ε_vM_ = 330) at 285 °C as well as by additional heat treatments at 60–150 °C in a silicon oil bath.

The use of warm HPT plus subsequent thermal treatment as a processing tool is beneficial with respect to several features:

(i) re-solutionizing of primary precipitates being possible sources of corrosion and brittleness;
(ii) generation a high number of dislocations; those account for the major part i.e., for more than 70% of the whole HPT-induced increase of strength (being 55%); 7% of it can be ascribed to the decrease of grain size. The considerable ductility (15%) can be related to the low level of internal stresses, thanks to near-equilibrium arrangements of dislocations achieved by the high HPT-processing temperature.
(iii) extra hardening (at least 20%) coming from thermal treatment allowing for the agglomeration of deformation induced vacancies;
(iv) precipitates that contribute also more than 20% to the total strength are present after HPT at the latest; however, they do not change during thermal treatment and thus do not contribute to the related hardening, nor decrease ductility;
(v) although there have been indications of dynamic and static recrystallization in the materials’ textures, they did not significantly affect the values of Young’s modulus, nor the experimental or the calculated values;
(vi) thanks to the elimination of primary precipitates, the corrosion rates could be decreased by the current processing route. Neither the generation of dislocations nor that of vacancy agglomerates had a marked impact on corrosion rates.

These features suggest the materials and especially the described processing route to be very appropriate for biodegradable implant materials.

## Figures and Tables

**Table 1:** Chemical composition of the investigated alloys.

Alloy	Mg [wt %]	Zn [wt %]	Ca [wt %]	Zr [wt %]
Mg5Zn0.3Ca	94.28 ± 0.03	5.44 ± 0.03	0.28 ± 0.03	/
Mg5Zn	94.77 ± 0.03	5.23 ± 0.03	/	/
Mg5Zn0.15Ca	94.9 ± 0.03	5.1 ± 0.03	0.15 ± 0.03	/
Mg5Zn0.15Ca0.15Zr	94.4 ± 0.03	5.6 ± 0.03	0.18 ± 0.03	0.18 ± 0.03

**Table 2:** Microhardness values of all investigated alloys in all conditions. HPT has been performed at 285 °C.

Sample	Condition	HV0.05
Mg5Zn0.3Ca	as-cast	65 ± 6
	HPT	99 ± 3
	HPT + HT 100 °C, 24 h	123 ± 6
	HPT + HT 150 °C, 2 h	119 ± 12
Mg5Zn	as-cast	70 ± 9
	HPT	95 ± 3
	HPT+HT 100 °C, 24 h	114 ± 10
	HPT+HT 150 °C, 2 h	106 ± 6
Mg5Zn0.15Ca0.15Zr	as-cast	79 ± 5
	HPT	101 ± 12
	HPT+HT 100 °C, 24 h	125 ± 6
	HPT+HT 150 °C, 2 h	123 ± 4

**Table 3:** Measured yield strength σ_yield_ and ultimate tensile strength (UTS), as well as ductility (total elongation) ε_total_ for all tested alloys in all conditions (‘HPT’ at 4 GPa, 10 rotations at 285 °C and heat treatment ‘HT’ at 100 °C for 24 h).

Sample	Condition	σ_yield_ [MPa]	UTS [MPa]	ε_total_ [%]
Mg5Zn0.3Ca	HPT	223 ± 34	248 ± 37	18 ± 2
Mg5Zn0.3Ca	HPT + HT	220 ± 33	249 ± 37	17 ± 2
Mg5Zn	HPT	156 ± 23	215 ± 32	17 ± 2
Mg5Zn	HPT + HT	170 ± 26	245 ± 37	19 ± 2
Mg5Zn0.15Ca	HPT	236 ± 35	270 ± 41	16 ± 2
Mg5Zn0.15Ca	HPT + HT	226 ± 34	250 ± 38	19 ± 2
Mg5Zn0.15Ca0.15Zr	HPT	226 ± 34	283 ± 42	19 ± 2
Mg5Zn0.15Ca0.15Zr	HPT + HT	270 ± 41	283 ± 42	16 ± 2

**Table 4:** Young’s modulus E measured by indentation microhardness of the alloys after HPT-processing by 10 rotations at 285 °C, and after additional heat treatment at 100 °C for 24 h. Simulated values E_sim_ are given in parentheses.

Sample	Condition	E (E_sim_) [GPa]
Mg5Zn0.3Ca	HPT	38 ± 2 (45)
Mg5Zn0.3Ca	HPT + HT	37 ± 1 (45)
Mg5Zn	HPT	34 ± 1
Mg5Zn	HPT+HT	34 ± 2
Mg5Zn0.15Ca	HPT	35 ± 1 (58)
Mg5Zn0.15Ca	HPT+HT	31 ± 1 (60)
Mg5Zn0.15Ca0.15Zr	HPT	38 ± 2
Mg5Zn0.15Ca0.15Zr	HPT+HT	42 ± 1

**Table 5:** Measured and calculated data for DSC peak I. ρ is the dislocation density (measured), E_disl_ is the dislocations’ stored energy calculated from ρ, and c_v_ is the vacancy concentration (calculated).

Sample	E_total_ [J/g]	*ρ* [10^14^ m^−2^]	E_disl_ [J/g]	E_vac_ [J/g]	c_v_ [10^−4^]
Mg5Zn	2.4 ± 0.5	2.5 ± 0.1	0.01 ± 0.001	2.4 ± 0.3	7 ± 1.0
Mg5Zn0.3Ca	1.9 ± 0.43	3.0 ± 0.1	0.1 ± 0.01	1.8 ± 0.4	5 ± 1.0
Mg5Zn0.15Ca	2.7 ± 0.13	3.6 ± 0.1	0.04 ± 0.01	2.7 ± 0.2	8 ± 0.6
Mg5Zn0.15Ca0.15Zr	2.6 ± 0.1	2.5 ± 0.1	0.01 ± 0.001	2.6 ± 0.04	8 ± 0.1

**Table 6:** Measured and calculated data for DSC peak II. ρ is the dislocation density (measured), E_disl_ is the dislocations’ stored energy calculated from ρ, and c_v_ is the vacancy concentration (calculated).

Sample	E_total_ [J/g]	*ρ* [10^14^ m^−2^]	E_disl_ [J/g]	E_vac_ [J/g]	c_v_ [10^−6^]
Mg5Zn	0.6 ± 0.08	3.8 ± 0.1	0.5 ± 0.06	0.07 ± 0.01	20 ± 6.0
Mg5Zn0.3Ca	0.4 ± 0.08	3.3 ± 0.1	0.4 ± 0.04	0.02 ± 0.01	6 ± 1.0
Mg5Zn0.15Ca	0.4 ± 0.1	3.5 ± 0.1	0.4 ± 0.1	0.01 ± 0.001	3 ± 1.0
Mg5Zn0.15Ca0.15Zr	0.4 ± 0.1	4.5 ± 0.1	0.4 ± 0.06	0.01 ± 0.001	0.6 ± 0.1

## Data Availability

Source data necessary to reproduce the results reported in this paper cannot be shared at the time of publication since they also form a part of an ongoing study. However, they can be made available upon request.
